# 

**Published:** 1899-06

**Authors:** 


*The following donations have been received since the publication of the list in March:*

*May 31st, 1899.*

American Dermatological Association (1) ... ... 10 volumes.
Dundee Medical Library (2) ... ... ... ... ... 1 volume.
L. M. Griffiths (3) ... ... ... ... ... ... ... 7 volumes.
Editor of *Illustrirte Rundschau der medicinisch-chirurgischen Technik* (4) ... ... ... ... ... ... ... 1 volume.
G. T. Jackson, M.D. (5) ... ... ... ... ... ... 1 ,,
E. D. Mapother, M.D. (6) ... ... ... ... ... ... 1 ,,
R. Shingleton Smith, M.D. (7) .. ... ... ... .. 1 ,,
E. H. E. Stack, M.B. (8) ... ... ... ... ... ... 1 ,,
Surgeon-General, United States Army (9) ... ... 29 volumes.
B. W. Walker, M.D. (10) ... ... ... ... ... ... 18 ,,
John Wright and Co. (11) ... ... ... ... ... ... 1 volume.

Unbound periodicals have been received from Mr. L. M. Griffiths. A framed picture has been presented by Mrs. Greig Smith; and unframed pictures have been given by Mr. H. Kerslake, Herr J. F. Lehmann, and Dr. William Warren Potter.

### THIRTY-SECOND LIST OF BOOKS.

The titles of books mentioned in previous lists are not repeated.

The figures in brackets refer to the figures after the names of the donors, and show by whom the volumes were presented. The books to which no such figures are attached have either been bought from the Library Fund or received through the *Journal.*

**Adams, J. H.** ... *Life of D. Hayes Agnew* ... 1892

**Adams, R.** ... *Illustrations of Rheumatic Gout* ... (8) 1857

**Allbutt, T. C.** [Ed.] *A System of Medicine.* Vol. VI. ... 1899

**Bailey, J. B.** ... *The Diary of a Resurrectionist,* 1811–12 ... 1896

**Barbour, A. H. F.** *The Anatomy of Labour* ... 2nd Ed. 1899

**Bettany, G. T.** ... *Eminent Doctors.* 2 vols. ... 2nd Ed. [1885]

**Bramwell, B.** ... *Anæmia and Diseases of the Blood-Forming Organs and Ductless Glands* ... 1899

**Brockbank, E. M.** *The Murmurs of Mitral Disease* ... 1899

**Brown, A. M.** ... *Elements of Alkaloidal Ætiology* ... 1899

**Bruce, J. M.** ... *Materia Medica and Therapeutics* ... [5th Ed.] 1896

**Bryce, J.** ... *On the Inoculation of Cowpox* ... 2nd Ed. 1809

**Burghard, W. W. Cheyne and F. F.** *A Manual of Surgical Treatment.* Part I. ... 1899

**Cheyne and F. F. Burghard, W. W.** *A Manual of Surgical Treatment.* Part I. ... 1899

**Crile, G. W.** ... *Surgical Shock* ... [1899]

**De Foe, D.** ... *A Journal of the Plague Year* ... [N.D.]

**Evans, A. H.** ... *Golden Rules of Medical Practice* ... [1899]

**Feltoe, Rev. C. L.** *Memorials of John Flint South* ... (7) 1884

**Fernie, W. T.** ... *Animal Simples* ... 1899

**Floderus, B.** .. *Om Medfödda Uretermissbildningar ur Kirurgisk Synpunkt* ... 1899

**Freeman, E. C.** ... *The Sanitation of British Troops in India* ... 1899

**Hawthorne, C. O.** *Some Aspects of the Mental Discipline Associated with the Study of Medicine* ... 1899

**Helferich, H.** ... *On Fractures and Dislocations.* (Tr. from 3rd Ed. by J. Hutchinson, jun.) ... 1899

**Herschell, G.** ... *Constipation* ... 1899

**Hime, M. C.** ... *Schoolboys' Special Immorality* ... 1899

**Hurst, A. M. Marshall and C. H.** *Practical Zoology.* 5th Ed. (Revised by F. W. Gamble) ... 1899

**Jackson, G. T.** ... *A Dermatological Bibliography* ... (5) 1891

*Katalog over det Norske Medicinske Selskabs Bibliotek—Tillægs No. 1* ... 1899

**Kirkes, [W. S.]** ... *Hand-Book of Physiology.* 15th Ed. (Ed. by W. D. Halliburton) ... 1899

**Leeuwenhoek, A. à** *Opera Omnia.* 4 vols. ... Editio Novissima 1719-22

**Mapother, E. D.** ... *Papers on Dermatology, &c.* ... (6) 2nd Ed. 1899

**Marshall and C. H. Hurst, A. M.** *Practical Zoology.* 5th Ed. (Revised by F. W. Gamble) ... 1899

**Moure, E. J.** ... *De la Réunion immédiate du Pavillon de l'Oreille après la Cure radicale de l'Otorrhée* ... ... ... ... 1899

" ... *Sur un Cas d'Ostéo-Myélite Aiguë du Temporal consécutive à l'Influenza* ... ... ... ... ... ... ... 1899

**Newsholme, A.** ... *Vital Statistics* ... ... ... ... ... ... 3rd Ed. 1899

**Ogle, J. W.** ... *The Harveian Oration, 1880* ... ... ... ... ... ... 1881

**Osler, W.** ... ... *The Principles and Practice of Medicine* ... ... 3rd Ed. 1898

**Park, R.** ... ... *An Epitome of the History of Medicine* ... ... 2nd Ed. 1899

**Parke, T. H.** ... *Experiences in Equatorial Africa* ... ... ... ... ... 1891

**Pedley, R. D.** ... *The Hygiene of the Mouth* ... ... ... ... ... ... [N.D.]

**Prichard, J. C.** ... *The Natural History of Man.* (Ed. by E. Norris) 2 vols. ... ... ... ... ... ... ... 4th Ed. 1855

" ... *Celtic Nations.* (Ed. by R. G. Latham) ... ... ... 1857

**Ratzel, F.** ... ... *The History of Mankind.* (Tr. from 2nd German Ed. by A. J. Butler) 3 vols. ... ... ... 1896-98

**Raynalde, T.** ... *The Byrth of Mankinde* ... ... ... ... ... ... ... 1604

**Roberts, C.** ... ... *Eastbourne as a Health Resort* ... ... ... ... ... ... 1899

**Rolleston, H. D.** *The Diseases and Primary Tumours of the Thymus Gland* [N.D.]

**Rosenberg, A.** ... *Die Krankheiten der Mundhöhle, des Rachens und des Kehlkopfes* ... ... .. ... Zweite Auflage 1899

*Sandefjord Svovl- og Sobad i Norge* ... ... ... ... ... ... ... ... ... 1899

**Savill, T. D.** ... *Neurasthenia* ... ... ... ... ... ... ... ... ... 1899

*Schools, A Code of Rules for the Prevention of Infectious and Contagious Diseases in* ... ... ... ... ... ... 4th Ed. 1899

**Simpson, J. Y.** ... *Syphilis in Scotland in the 15th and 16th Centuries* ... [1862]

**Sisley, R.** ... ... *The London Water Supply* ... ... ... ... ... ... 1899

**Smith, E. N.** ... *Growing Children: Their Clothes—and Deformity* ... 1899

**Smith, F. W.** ... *The Natural Waters of Harrogate* ... ... ... ... ... 1899

**Spitta, E. J.** ... *Photo-Micrography* ... ... ... ... ... ... ... ... 1899

*Therapeutics of Tuberculosis, Bronchitis, and Scrofula* ... ... ... ... ... [N.D.]

**Thompson, C. J. S.** *The Mystery and Romance of Alchemy and Pharmacy* ... 1897

" ... *Poison Romance and Poison Mysteries* ... .. ... ... 1899

**Tillmanns, H.** ... *A Text-Book of Surgery.* Vols. I. (Tr. from 3rd German Ed. by J. Rogers and B. T. Tilton. Ed. by L. A. Stimson), II., III. (Tr. from 4th German Ed. by B. T. Tilton. Ed. by L. A. Stimson) ... ... ... ... ... ... ... 1898-99

**Tuchmann, M.** ... *The Exploration of the Urethra and Bladder* ... ... ... 1899

**Turner, R.** ... ... *Lord Lister and Surgery* ... ... ... ... ... ... ... 1899

**Verworn, M.** ... *General Physiology.* (Tr. from 2nd German Ed. and Ed. by F. S. Lee) ... ... ... ... ... 1899

**Whitelegge, B. A.** *Hygiene and Public Health* ... ... ... [8th Ed.] 1899

**Williams, C. J. B.** *Memoirs of Life and Work* ... ... ... ... ... ... 1884

---

### TRANSACTIONS, REPORTS, JOURNALS, &c.

Alienist and Neurologist, The... ... ... ... ... ... ... ... Vol. XIX. 1898

American Dermatological Association, Transactions of the (1) 1878, 1879, 1886, 1889-93; 1896-98

American Gynæcological and Obstetrical Journal, The Vols. XI., 1897, XIII., 1898

American Journal of Obstetrics, The ... ... ... ... Vol. XXXVIII. 1898
American Journal of Ophthalmology, The ... ... ... ... Vol. XV. 1898
American Ophthalmological Society, Transactions of the... Vol. VIII., Part 2 1898
American Pediatric Society, Transactions of the ... ... ... Vol. X. 1898
American Practitioner and News, The... ... ... Vols. XXV., XXVI. 1898
American Year-Book of Medicine and Surgery, The... ... ... ... ... 1899
Annales de la Société Belge de Chirurgie ... ... ... ... Tome VI. 1898
Annali di Ostetricia e Ginecologia... ... ... ... ... ... ... ... ... 1898
Annual and Analytical Cyclopædia of Practical Medicine ... Vol. III. 1899
Archiv für Verdauungs-Krankheiten ... ... ... ... ... Band IV. 1898
Archives of Surgery ... ... ... ... ... ... ... ... ... ... ... Vol IX. 1898
Archives provinciales de Chirurgie ... ... ... ... ... Tome VII. 1898
Australasian Medical Gazette, The ... ... ... ... ... Vol. XVII. 1898
Bristol and Clifton Directory ... ... ... ... ... ... ... ... ... ... (11) 1899
Bristol Medico-Chirurgical Journal, The ... ... ... ... Vol. XVI. 1898
Buffalo Medical and Surgical Journal, The... (9) Vols. XX.—XXVI. 1881-87
Bulletin de l'Académie de Médecine 3e Sér., Tomes XXXIX., XL. 1898
Bulletin de l'Académie royale de Médecine de Belgique ... Tome XII. 1898
Canadian Practitioner, The ... ... ... ... ... ... ... Vol. XXIII. 1898
Centralblatt für Chirurgie ... ... ... ... ... ... ... ... 1888, 1898
Centralblatt für Gynäkologie ... ... ... ... ... ... ... ... 1877-92, 1898
Centralblatt für innere Medicin ... ... ... ... ... ... ... ... ... 1898
Cincinnati Lancet and Clinic, The ... ... ... (9) Vols. XL.—LI. 1878-84
Cincinnati Lancet-Clinic, The ... ... ... ... ... ... Vol. LXXX. 1898
College of Physicians of Philadelphia, Transactions of the 3rd Ser., Vol. XX. 1898
Dublin Journal of Medical Science, The (3) Vols. LXIX., 1880, LXXI. 1881
Écho médical du Nord, L' ... ... ... ... ... ... ... ... ... ... 1898
Food Journal, The ... ... ... ... ... ... ... ... (3) Vols. I.—IV. 1871-74
France médicale, La ... ... ... ... ... ... ... ... ... ... ... ... ... 1898
Gazette des Hôpitaux de Toulouse ... ... ... ... ... ... 1897, 1898
Gazette hebdomadaire des Sciences médicales de Bordeaux Tome XIX. 1898
Gazzetta Medica Lombarda ... ... ... ... ... ... ... ... ... ... ... 1898
General Medical Council, Minutes of the ... ... ... ... Vol. XXXV. 1899
Hospitals—
Middlesex Hospital, The—Registrars' Reports for 1897 ... ... 1898
Illustrirte Rundschau der medicinisch-chirurgischen Technik ... (4) 1898
Intercolonial Medical Journal of Australasia ... ... ... ... Vol. III. 1898
International Medical Magazine ... ... ... ... ... ... Vol. VII. 1898
Journal d'Hygiène ... ... ... ... ... ... ... ... ... ... Vol. XXIII. 1898
Journal of Laryngology, Rhinology, and Otology, The ... Vol. XIII. 1898
Journal of Mental Science, The Vols. (10) XIX.—XXII., 1874-77; (2) XXV., 1880; (10) XXVI.—XXXVII., 1881-91, XXXIX., 1893, XLI., 1895
Journal of the American Medical Association, The ... ... Vol. XXXI. 1898
Laryngoscope, The ... ... ... ... ... ... ... ... ... ... Vols. IV., V. 1898
Library, The ... ... ... ... ... ... ... ... ... ... ... ... ... Vol. X. 1898
Liverpool Medico-Chirurgical Journal, The ... ... ... Vol. XVIII. 1898
Medical Age, The ... ... ... ... ... ... ... ... ... ... Vol. XVI. 1898

| | | |
|---|---|---|
| Medical Annual, The | | 1899 |
| Medical Chronicle, The | N.S., Vol. X. | 1898-99 |
| Medical Defence, A Year's Work in (1898) | | [1899] |
| Medical Officer of the Privy Council, 11th Report of the | (3) | 1869 |
| Montreal Medical Journal, The | Vol. XXVII. | [1898] |
| Newquay Urban District Council—Annual Report of the Medical Officer of Health | | 1898 |
| New York Journal of Gynæcology and Obstetrics, The | Vols. I., II. | 1891-92 |
| New York Medical Journal, The (9) Vols. V., 1867, IX.—XII., 1869-70, XIV., 1871, XIX.—XXII., 1874-75; | LXVIII., | 1898 |
| Nord médical, Le | Tome IV. | 1898 |
| Northumberland and Durham Medical Journal, The | | 1898 |
| Nursing Directory, Burdett's Official | | 1899 |
| Occidental Medical Times | Vol. XII. | 1898 |
| Post-Graduate, The | Vol. XIII. | [1898] |
| Preparations, Annual Report on New Medicinal, 1898 | | 1899 |
| Progrès médical, Le | 3e Sér., Tome VIII. | 1898 |
| Revue de Thérapeutique médico-chirurgicale | 1896, | 1898 |
| Revue génèrale d'Ophtalmologie | Tome XVII. | 1898 |
| Revue hebdomadaire de Laryngologie, d'Otologie, et de Rhinologie | Tome XVIII. | 1898 |
| Revue médicale, La | | 1898 |
| Revue médico-chirurgicale des Maladies des Femmes | Tome XIX. | 1897 |
| Sanitary Record, The | Vol. XXII. | 1898 |
| Society of Medical Officers of Health, Transactions of the | | 1885-87 |
| Therapeutic Gazette, The | Vol. XXII. | 1898 |
| Treatment | Vol. II. | 1899 |
| Wiener klinische Wochenschrift | | 1898 |
| Zeitschrift für Krankenpflege | | 1898 |

### Local Medical Notes.

### ABERGAVENNY.

Monmouthshire Asylum.—During the year 1897 this asylum increased the number of its patients by 37, and on the last day of the year it contained 1,012. Of the year's admissions 18 owed their insanity to moral causes, 21 to intemperance in drink, 45 to hereditary influence, and 42 to previous attacks. Courses of lectures on nursing are given by the medical officers, and eighteen of the staff entered for the examination of the Medico-Psychological Association, and seventeen of them obtained the nursing certificate. The committee express their "high appreciation of the services of Dr. Glendinning and his able assistants, Dr. Nelis and Dr. Black." At the meeting of the Monmouthshire County Council, held on May 3rd, it was decided to purchase at an approximate cost of £8,000 a farm adjacent to the county asylum for purposes of further development.

### BARNSTAPLE.

Public Health.—Mr. M. R. Gooding has been re-appointed Medical Officer to the Port Sanitary Authority.

### BATH.

Royal United Hospital.—At the Guildhall, Bath, under the presidency of the Mayor, the annual meeting of the Governors and others interested in the Bath Royal United Hospital was held. According to the report the number of in-patients that had been treated was 1,190, whilst in 1897 the number was 1,264. The out-patients numbered 8,209, whilst during the previous year the number was 8,988. An increase of subscriptions from factories and workshops was shown; but there had been a falling off in subscriptions and donations during the past year. Several improvements have been effected, the operating theatre and the new chapel having been refurnished and kitchens constructed.

Eye Infirmary.—At the annual meeting of the Governors of the Bath Eye Infirmary, the report showed that against 262 in-patients treated during 1897, 208 were admitted during the past year. £245, the amount of legacies received, and the balance in hand, which was a good one, had been carried to the capital account.

Royal Mineral Water Hospital.—The annual meeting of this institution was held on May 1st, under the presidency of General Mainwaring. The report showed that during the past year 1350 patients had been admitted, the largest number in the history of the hospital; the average number in the hospital was 150, and the average stay of each was 40 days. The expenditure amounted to £5,514 and exceeded the income by £142. The balance in hand was £527. Colonel Vaughan was elected president for the ensuing year.

### BRISTOL.

Hospital Sunday Fund.—In our last number we stated that the collection this year on behalf of the Medical Charities would exceed that of 1898, and we are pleased to say that about £500 more was obtained. This must be very gratifying to the organisers, who certainly worked very hard to make the fund a success. In only the second year of its existence, that the collections in the numerous places of worship in our city should contribute £1500 is good, but when compared with what is accomplished in other towns it is evident that much remains to be done to stimulate interest in our medical charities and their impoverished condition. Comparing the lists of last year and of this, it is a curious fact, and one of which no explanation has yet been given, that while the money received from Church of England churches is less than previously, that from Nonconformists has increased. It may be that the Nonconformist conscience has been awakened; if so, we hope that the Established conscience will be awakened next year.

Annual Reports of Medical Charities. — Since March all the Medical Charities have published their annual reports, and in all of them there is the same cry for more subscribers to carry on the work. The ever increasing debt of these institutions is a very serious matter, and must be faced. Several of them have employed legacies to pay current expenses—a proceeding which does not meet with the approval of everyone; but when the income year by year fails to meet the expenditure, something of this kind must be done.

The Prevention of Consumption.—The Committee mentioned in our last number have been at work, but not much progress has been made beyond a most excellent report on the condition of the city from a medical and legal point of view presented by Dr. Davies. We believe that this will be printed in the Medical Officer's annual report, so that at least every medical man as well as town councillor will have an opportunity of seeing how much consumption there is in Bristol now as compared with forty years ago; and how we are still in a very impotent state as regards the inspection of meat or dairies. We regret that so little interest has been shown outside our profession in the matter, but it may be aroused later.

The Jubilee Convalescent Home will be opened, according to present arrangements, by the Queen in November. The Committee has got so far as to elect a Medical Officer, and out of a large number of candidates chose Mr. John S. Griffiths for the post. The interior is still undergoing alterations.

Obituary.—It is with deep regret that we learn that the plague in India has caused the death of Dr. Fenton Evans, who was some years ago a very able Medical Tutor at the School, and afterwards House Physician at the Royal Infirmary.

### BURNHAM.

Public Health. — Dr. F. C. Berry has been re-appointed Medical Officer of Health.

### CARDIFF.

Public Health.—Dr. E. M. Spencer has been appointed Medical Officer to the Penarth Sanitary District. Dr. R. Mathias has been appointed Medical Officer for the Pentyrch Sanitary District.

The Infirmary.—At a meeting of the Cardiff Infirmary, held on April 12th, under the presidency of Dr. Edwards, it was decided that a sub-committee should report upon the proposed reception of paying patients in the institution. A recommendation of the medical board that no member of the honorary staff should hold office longer than twenty years was deferred for future consideration. It was decided that the supply of milk to the infirmary should be only from cows certified to be free from tuberculosis. Legacies to the amount of £2,100 have been recently received by the institution, and the reverse balance is now reduced to £2,900. A resolution was proposed at a special meeting of the governors that a deputation should approach the Mayor and County Council of Cardiff with a view to obtaining a grant of not less than £500 a year from the Council to the Infirmary towards maintenance expenses; but the resolution was rejected by a majority of ten.

Proposed Seamen's Hospital.—The Mayor of Cardiff has embarked upon a useful and praiseworthy scheme; namely, that of building and organising a Seamen's Hospital for the town. Already the sum of £16,500, including a generous donation of £10,000 from Lord Bute, has been raised, but £5,500 more are required before the work can be proceeded with. Cardiff is a wealthy town, and there is every probability that this sum will soon be forthcoming. Moreover, the object is so good a one that there can be no excuse for those able to do so from withholding their liberal support. The important shipping centre which the town has now become makes the needs of a Seamen's Hospital almost imperative, and hence the activity and enthusiasm displayed by the townspeople in the Mayor's enterprise. It is proposed to hold a bazaar in aid of the building fund in December next. We wish the new charity every success.

Convalescent Home.—The annual meeting of those interested in this institution was held at the Cardiff Town Hall on April 24th, under the presidency of the Mayor, Alderman T. Morel. The report showed that the receipts in 1898 were £1,453, and the expenditure amounted to £1,108. There still remains a deficit balance of £2,500 on the extension fund. There were 738 patients admitted, the average period for which each remained in the Home being seventeen days. The cost of maintenance was 1s. 5d. per head per day. It was decided that a special committee should be appointed to devise means for raising funds to remove the debt upon the building.

District Nursing.—The ninth annual report of the Cardiff Branch of Queen Victoria's Jubilee Institute for Nurses has been issued. The Honorary Treasurer, Dr. Sheen, reports that the total cost of this excellent work was £883 9s. 6d., which was raised by various methods.

### CARMARTHEN.

Public Health.—The decision at the last quarterly meeting of the Carmarthenshire County Council to set up a Sanitary Committee, to whom is delegated the question of securing the appointment of a County Medical Officer of Health, is a long delayed step which will eventually bring this County Authority in line with its compeers throughout the country in matters of public health.

Obituary.—The death of Mr. James Rowlands in his eighty-fifth year occurred on April 10th. He received his medical training at St, George's Hospital, London, and was believed to be the oldest St. George's man living. For forty years he was surgeon to the prison at Carmarthen, and for many years also surgeon to the local infirmary. He was Coroner for a long period, and an Alderman and J.P. He was deeply respected and beloved by all classes of the community, and remained in practice until illness compelled him to retire about six months ago. He married in 1835, and celebrated his diamond wedding in January, 1895.

### CHELTENHAM.

Public Abattoirs.—The Medical Officer of Health for Cheltenham, Dr. J. H. Garrett, refers to the abattoirs question in his report for 1898. He asserts that it is not only nor chiefly to avoid a nuisance from filth and general insanitary conditions, such as may be prevented to a great extent by by-laws, that private slaughter-houses should give way to public abattoirs, but rather in order that the very necessary inspection of animals intended for slaughter, and of their organs and carcasses after slaughter, and of their mode of slaughter, should be rendered possible, and the seizure or setting aside of the flesh and parts of diseased animals be facilitated.

Tuberculosis.—Dr. Garrett points out that it would be misleading to the public to grant a Corporation certificate, which would be taken as a guarantee of the freedom of the products of a dairy from the germs of tuberculosis, whilst in reality our knowledge of the subject does not at present admit of the possibility of any such guarantee. This has prevented him hitherto advising the use of certificates in his district.

### DEVIZES.

Public Health.—Dr. Leonard Raby has been appointed Medical Officer of Health, *vice* Mr. E. N. Carless, deceased. Mr. J. T. Thomas has been appointed Medical Officer of Health for Wiltshire.

### DEVONPORT.

Public Health.—Mr. H. Gard has been appointed Medical Officer for the Northern Sanitary District.

Royal Albert Hospital.—A scheme has been prepared for extending and improving the Royal Albert Hospital at an estimated cost of £4,000.

Isolation Hospital.—The Devonport Town Council at their meeting on April 13th decided to endeavour to purchase seven acres of land adjoining the Infectious Diseases Hospital, which was built in 1882, and is in need of enlargement.

### EXETER.

Devon and Exeter Hospital.—The special meeting of Governors of the Devon and Exeter Hospital dealt with a large number of questions of vital interest to all institutions of the kind. The Devon and Exeter Hospital has experienced a large amount of criticism recently, and a complete inquiry into its financial condition has taken place. At the meeting it was proposed to close one large ward, to decrease the yearly deficit. Discussion brought out the fact that the capital of the Hospital equalled upwards of £100,000, from which a yearly income of £3,000 was derived. In the face of this fact, a resolution in favour of resorting to other means of diminishing the adverse balance was passed, and it was also decided that a reinvestment of the capital should be considered. A proposal to open a paying ward did not receive much attention, and another suggestion for the more satisfactory admission of patients met with no definite result. Nevertheless, the meeting showed that amongst the governing body a distinct advancement has been made in realising the measures which tend generally towards progress and judicious administration.

West of England Eye Infirmary.—The foundation stone of the new West of England Eye Infirmary was laid by Mr. Franklin on April 11th, after a short service by the Bishop of Crediton. This infirmary was founded in 1808, and was the second in the United Kingdom devoted exclusively to diseases of the eye. In 1895 it was decided to entirely rebuild the institution, and property adjoining the infirmary was purchased for that purpose. The section now commenced will cost about £8,500, towards which £7,500 has been raised.

The Prevention of Consumption.—A useful leaflet, entitled *Advice on the Prevention of Consumption*, has just been issued for circulation among the people by the Health Department of Exeter, of which Dr. John Woodman is the Medical Officer of Health, and Drs. Edward A. Brash, George T. Clapp, and John Mackeith the Assistant Medical Officers.

### EXMOUTH.

Dispensary. — Dr. J. Shapland has been appointed Medical Officer, *vice* Mr. Reginald Martyn, resigned.

### FALMOUTH.

Public Health.—Dr. W. K. Bullmore has been re-appointed Medical Officer of Health to the Falmouth and Truro Port Sanitary Authority.

### GLOUCESTER.

Public Health.—The report of the Medical Officer of Health (Dr. Campbell) of the Gloucester Urban Sanitary Authority for 1898 has just been issued. Dr. Campbell estimates the total population of the city at 41,450, an increase of 284 in the year. The births numbered 1315—688 males and 627 females—compared with 1336 in the previous year. The rate is 31·7 per 1000, compared with 32·6 per 1000. There were 694 deaths as compared with 726, a rate of 16·7 as compared with 17·6 in the previous year. Dealing with the water supply, the report states that the three sources of supply now available are capable of providing twenty gallons per head per day for double the population now supplied. On the whole, the supply generally will, it is stated, compare favourably with that of the best supplied towns; and the only fault that can be found is that the Witcombe and Robinshood Hill supplies are not sand filtered as well as decanted, and that the Newent supply is not chemically softened.

Gloucester General Infirmary and Gloucester Eye Institution.—At a quarterly meeting of this institution, held on March 30th, under the presidency of Colonel Curtis Hayward, the Chairman stated that during the past ten years the expenditure of the Infirmary had increased by £1,000 a year, while the income had only increased by £200.

District Nursing.—The annual meeting of the Gloucester District Nursing Society has been held, under the presidency of the Dean. There was a good attendance, and the novelty of the proceedings was an address on the subject of "District Nursing" by Mrs. Clare Goslett. The Chairman, in addressing the meeting, remarked that general approval was sometimes not so profitable as a little wholesome abuse. Everyone approved of this most needful and helpful institution, but at the present moment they were from £30 to £40 to the bad. It has been decided to buy a house for the use of the nurses. The hon. secretary's report also contained a strenuous appeal for a larger income. The committee deplore the loss, by death, of Dr. Ancrum, vice-president of the Society since its institution, and of Miss Taynton, an energetic colleague.

### ISLES OF SCILLY.

Medical Magistrate.—Dr. T. Thornton Macklin, Medical Officer of Health for Scilly, has been appointed to the Commission of the Peace for the Isles of Scilly in the County of Cornwall.

### KEYNSHAM.

Public Health.—Mr. F. W. S. Stone has been appointed Medical Officer for the Sanitary District of Kelston and Northstoke.

### LOOE.

Public Health.—Dr. J. E. Webb has been re-appointed Medical Officer of Health.

### MAESTEG.

Action for Libel.—At the Glamorganshire Assizes, held on March 21st, before Mr. Justice Darling, a libel action was brought against Dr. John Davies, of Maesteg, by Dr. W. H. Thomas, J.P., of the same place. The cause of the action was the publication of a letter to the Bridgend Board of Guardians and also to a local newspaper, in which defendant complained of the absence of professional etiquette on the part of the plaintiff. The plaintiff claimed £1,000 damages. Eventually the defendant withdrew absolutely the statement which he had made against Dr. Thomas. In the result a juror was withdrawn.

We regret to record the sudden death of Dr. Thomas on April 13th.

### MERTHYR-TYDVIL.

Public Health.—The Medical Officer of Health (Mr. Dyke) reports that during 1898 there were 2495 births registered, giving a rate of 34·7; of these 73, equivalent to a rate of 2·92, were illegitimate. Vaccinations were performed in 1870 instances by public vaccinators. There were 1409 deaths, compared with 1598 in 1897, equal to a rate of 19·5 per 1000, which is the lowest rate ever recorded in the district. The present population is 71,903, with 12,912 occupied houses.

### MIDSOMER NORTON.

Public Health.—Mr. A. H. Whicher has been re-appointed Medical Officer of Health.

### NEWPORT.

Public Health.—The Sanitary Committee of the Corporation has decided to continue for another term of five years the old system of dealing with street and trade refuse; viz., by tipping it on scavenging heaps instead of purchasing and erecting refuse destructors. Inquiries as to the taking of further land for cemetery purposes have brought in a valuation of ten acres of land belonging to Lord Tredegar at £3,750, in addition to the cost which would be imposed for drainage.

### NEWTON ABBOT.

Public Health.—Mr. H. Goodwyn has been re-appointed Medical Officer for the Islington Sanitary District. Mr. R. H. Grimbly has been re-appointed Medical Officer for the Ipplepen Sanitary District. Mr. A. E. Hayward has been appointed Medical Officer for the Teignmouth Sanitary District.

### PENZANCE.

Vaccination.—At the meeting of the Penzance Board of Guardians, held on April 13th, it was stated that £102 would be paid for vaccination fees in the western district of the union, as compared with £15 paid for the whole union in the corresponding quarter of last year. One of the members thought that the names of persons vaccinated by the public vaccinator should be published.

### PLYMOUTH.

Borough Asylum.—At a meeting of the Plymouth Town Council, held on May 8th, the report for 1898 of the Visiting Committee of the Plymouth Borough Asylum at Blackadown was presented. This stated that the result of the recent controversy between the Guardians and the Council with regard to the weekly rate charged for pauper patients from Plymouth was that the Council in future would pay £1000 a year extra and the Guardians £1000 per annum less than formerly. The weekly maintenance rate of patients had been reduced from 12s. 1d. to 11s. 2d.

Proposed Home for Incurables.—At an influential meeting, held at Plymouth on March 29th, it was decided to establish a Home for Incurable Patients at Plymouth, and a committee was appointed to prepare a scheme for the purpose to be submitted to a public meeting to be held shortly.

### PONTYPRIDD.

Public Health.—Mr. D. N. Morgan has been appointed Medical Officer to the Tonyrefail and Gilfach Sanitary District.

### PORTISHEAD.

Sewerage Scheme.—These works have been completed, and the house connections are being rapidly proceeded with. The sewage, without treatment of any kind, is discharged into the river Severn at a point about a third of a mile from the shore. Valves are provided to keep the sea water out of the sewers; and the low-level sewers are distinct from the high-level, the sewage having to be lifted from the former to the latter. The total length of main sewers on the high and low levels is about eight miles. The whole works cost, approximately, £16,500.

### SWANSEA.

Swansea Hospital.—Mr. C. N. Chadborn has been appointed House Surgeon.

Diphtheria Epidemic.—In the recent diphtheria epidemic at Swansea a good effect was produced by closing the elementary schools.

### TAUNTON.

Taunton and Somerset Hospital.—Dr. A. C. Anderson has been appointed Assistant House-Surgeon.

### TAVISTOCK.

Respiratory Disorders among Arsenic Workers.—Since the decline of the copper and tin mining industries in Cornwall, a number of arsenic works have been in operation on the sites of the old mines, and the attention of the Tavistock Board of Guardians has been directed to the large number of persons employed at these works who have been disabled and have come upon the parish for support. Dr. C. C. Brodrick, Medical Officer of Health for the Tavistock Rural District, and Dr. Albert Bowhay, Medical Officer of Health for the Calstock Rural District, at the request of the Board, have investigated the state of affairs in the parish of Calstock, and find that a large number of persons once employed at the arsenic works are in receipt of parish relief, and are suffering from respiratory diseases attributed to the effect of arsenic fumes. They have also discovered that out of 100 persons employed at these works who have died in the last three years, 83 have died of respiratory diseases. On visiting the works they observed that the furnace men used no protection at all, while the millers and grinders had the mouth and nostrils covered only with lint and a handkerchief. They recommend the use of a wire mask covered with gauze by all those employed at the works. The Board of Guardians have sent copies of this report to the managers of the works, and also to the Local Government Board. These two medical officers have for many years been familiar with the arsenic works, and are well acquainted with the medical history of the workers, so that their opinion is deserving of serious consideration. (For further information relating to this subject, see *British Medical Journal*, April 15th, 1899, p. 926.)

### TOTNES.

New Hospital.—At the meeting of the subscribers of the Totnes (Devon) Cottage Hospital, held on March 22nd, under the presidency of the Mayor (Dr. G. J. Gibson), the Duke of Somerset's offer of a site for a new hospital at Bridgetown was gratefully accepted, and it was decided to advertise for plans for the new building.

### TROWBRIDGE.

Public Health.—At a special meeting of the Urban Council held last week, consideration was given to the question of the proposed formation of an isolation hospital district for the urban district, the Melksham urban district, and the rural district of the Melksham union. A resolution against joining the hospital district was passed unanimously.

### TRURO.

Public Health.—At the meeting of the Sanitary Committee of the Cornwall County Council, held at Truro on April 13th, under the presidency of the Hon. John Boscawen, Mr. Martyn drew attention to the desirability of appointing a Medical Officer of Health for the County. The chairman agreed as to the necessity of such an appointment, and upon his motion a sub-committee was elected to act.

Tuberculosis.—The Medical Officer of Health for Truro Union (Dr. Bonar), in his report to the Sanitary Committee, recently referred at length to tuberculosis in cows, and stated that one cow's milk with tubercle mixed with the milk of twenty healthy cows would contaminate the whole. He also referred to the importance of the tuberculin test, and said it would be an advantage to every milk purveyor to be able to say that his dairy was free from suspicion. Commenting on the report, Dr. Bonar said he thought that all dairies from which milk was brought to Truro should have their cows tested. Those who lived by milk-selling ought to be able to say they had had their animals tested, and that these were free from tubercle. Dr. Bonar said the time was coming when no one in Truro or anywhere else would buy milk from cows that had not passed the test.

CORNWALL NURSING ASSOCIATION.—The Earl of Mount Edgcumbe presided at the annual meeting of the Cornwall County Nursing Association, recently held at Truro. There was a large and influential attendance. The village nurses, trained by the Association free of cost to themselves, have been started with a view to meet the want of trained nursing in scattered rural districts, where it is impossible to raise the sum required to maintain a Queen's nurse. The minimum time required for training village nurses is six months, but the Executive Committee have decided in some instances to allow twelve months instead, so as to better qualify them for their work. These nurses are required to pass the examination of the London Obstetrical Society, and to obtain its certificates. Arrangements have been made to give the village nurses six months' additional training in the South Devon and East Cornwall Hospital, Plymouth, and the Royal Cornwall Infirmary, Truro, subject to certain conditions. The Association has now altogether expended £229 on the training of nurses, and has received a grant of £100 from the Cornwall Technical Education Committee.

### WEYMOUTH.

PUBLIC HEALTH.—Mr. James Nimmo has been appointed Public Analyst to the Borough of Weymouth and Melcombe Regis.

### A Memorial to Dr. Joseph O'Dwyer.

A representative committee has been formed for the purpose of doing honour to the memory of Dr. Joseph O'Dwyer. Dr. Geo. F. Shrady has been elected permanent Chairman, and Dr. Alfred Meyer permanent Secretary, and the following Committee on Scope and Plan was appointed: Dr. Dillon Brown, Chairman, and Drs. Robert Abbe, R. G. Freeman, L. Emmet Holt, and Louis Fischer. The memorial will probably take an educational form.

### The "Index Medicus."

The announcement has just been made that with the current number the *Index Medicus* will cease to exist. The death of this periodical will be an irreparable loss, as during the twenty-one years of its able editorship it has become an indispensable necessity in the field of medicine. Some steps must be taken to continue it under under new conditions. We are not in a position to say how this can be done; but as the question is a cosmopolitan one, some scheme by which the great medical institutions and societies of all countries could become guarantors ought not to be an impossibility. Could not the Royal Colleges of the United Kingdom take the initiative in the matter?

